# Revealing a Pathway for Low‐Temperature Recrystallization in Germanium

**DOI:** 10.1002/advs.202507630

**Published:** 2025-08-14

**Authors:** Gihan Velişa, Eva Zarkadoula, Decebal Iancu, Maria D. Mihai, Alexandre Boulle, Yang Tong, Da Chen, Yanwen Zhang, William J. Weber

**Affiliations:** ^1^ Horia Hulubei National Institute for Physics and Nuclear Engineering IF Măgurele 077125 Romania; ^2^ Center for Nanophase Materials Sciences Oak Ridge National Laboratory Oak Ridge TN 37831 USA; ^3^ Institut de Recherche sur les Céramiques CNRS UMR 7315 Limoges 87068 France; ^4^ Institute for Advanced Studies in Precision Materials Yantai University Yantai Shandong 264005 China; ^5^ School of Energy and Environment Southeast University Nanjing 211189 China; ^6^ Department of Mechanical and Materials Engineering Queen's University Kingston Ontario K7L2N8 Canada; ^7^ Department of Materials Science & Engineering University of Tennessee Knoxville TN 37996 USA

**Keywords:** athermal recovery, complete damage annealing, defect analyses, defects simulation, germanium

## Abstract

Thermally activated annealing in semiconductors faces inherent limitations, such as dopant diffusion. Here, a nonthermal pathway is demonstrated for a complete structural restoration in predamaged germanium via ionization‐induced recovery. By combining experiments and modeling, this study reveals that the energy transfer of only 2.4 keV nm^−1^ from incident ions to target electrons can effectively annihilate pre‐existing defects and restore the original crystalline structure at room temperature. Moreover, it is revealed that the irradiation‐induced crystalline‐to‐amorphous (c/a) transformation in Ge is reversible, a phenomenon previously considered unattainable without additional thermal energy imposed during irradiation. For partially damaged Ge, the overall damage fraction decreases exponentially with increasing fluence. Surprisingly, the recovery process in preamorphized Ge starts with defect recovery outside the amorphous layer and a shrinkage of the amorphous thickness. After this initial stage, the remaining damage decreases slowly with increasing fluence, but full restoration of the pristine state is not achieved. These differences in recovery are interpreted in the framework of structural differences in the initial defective layers that affect recovery kinetics. This study provides new insights on reversing the c/a transformation in Ge using highly‐ionizing irradiation and has broad implications across materials science, radiation damage mitigation, and fabrication of Ge‐based devices.

## Graphical abstract

This study reports the discovery that energy transferred to electrons and atoms by highly ionizing ions results in a disorder‐order (amorphous‐crystalline) phase transformation in noncrystalline (amorphous) Ge. This discovery unlocks low‐temperature recrystallization pathways in Ge, offering a nonthermal strategy for fabricating Ge‐based devices that are temperature‐sensitive.

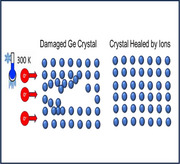

## 1 Introduction

Scientific and industrial interest in germanium (Ge) has surged over the past decade^[^ ^1^, ^2^, ^3^, ^4^, ^5^, ^6^ ^]^ fueled by the higher free charge carrier mobility and lower activation temperature for ion implanted dopants compared to that of silicon.^[^ ^7^ ^]^ These properties position Ge as a promising candidate for advanced semiconducting applications, such as high‐quality photodetectors,^[^ ^8^ ^]^ nanoscale transistors,^[^ ^9^ ^]^ and high‐efficiency anodes for lithium‐ion batteries.^[^ ^10^ ^]^ Most of these applications require ion implantation in Ge, an industrial fabrication process that often results in undesirable disorder or an amorphous layer.^[^ ^7^, ^11^, ^12^, ^13^ ^]^ While such amorphous layers can be beneficial to create ripple patterns,^[^ ^14^, ^15^, ^16^ ^]^ defects and amorphization typically degrade the optical and electrical properties of Ge, necessitating post‐implantation defect removal to restore the defect free state.^[^ ^17^ ^]^ Usually, the healing of defects can be achieved either by thermal or laser annealing processes,^[^ ^1^ ^]^ but these techniques face limitations in scalability and thermal budget constraints.

Interestingly, ion‐beam‐induced defect recovery, observed in semiconductors like Si,^[^ ^18^ ^]^ offers an alternative pathway. This phenomenon occurs under subsequent irradiation across a wide energy range: high‐energy (*E* > 1 MeV amu^−1^),^[^ ^19^, ^20^ ^]^ intermediate‐energy (≈1 keV amu^−1^ < *E* < 1 MeV amu^−1^),^[^ ^18^, ^21^ ^]^ and low‐energy (*E* ≤ 1 keV amu^−1^).^[^ ^22^ ^]^ High‐ and intermediate‐energy incident ions induce athermal recovery via electronic energy loss (*S*_e_), while low‐energy ions trigger thermally mediated recrystallization through the nuclear energy loss (*S*_n_). The latter, known as ion beam induced epitaxial crystallization (IBIEC), relies on defect migration and recombination at the amorphous/crystalline interface. Although IBIEC has been observed in Ge,^[^ ^23^, ^24^ ^]^ its requirement for elevated temperatures (> 500 K) renders it incompatible with low thermal budget processing, a critical hurdle for industrial adoption. Here, we demonstrate a low‐temperature ion‐beam annealing strategy that overcomes these limitations, enabling defect‐free Ge recovery without thermal compromise.

The ionization‐induced recovery observed at room temperature (RT) under swift heavy ion (SHI) irradiation is known as the SHI beam‐induced epitaxial crystallization (SHIBIEC) process.^[^ ^25^ ^]^ The SHIBIEC process has been reported in predamaged Ge under SHI irradiations (140 MeV Kr^[^ ^26^ ^]^ and 100 MeV Ag ions^[^ ^1^ ^]^) at RT. However, an increase in level of pre‐existing disorder (i.e., approaching preamorphized state) requires elevated irradiation temperatures (≥ 475 K),^[^ ^1^, ^2^ ^]^ being incompatible with low thermal budget processing. Additionally, this *S*_e_‐mediated athermal recovery has, to our knowledge, not been reported for intermediate energies, despite observations in other semiconductors (e.g., SiC^[^ ^27^ ^]^ and Si^[^ ^28^ ^]^). Complete recovery of pre‐existing defects in Ge with intermediate‐energy ions would advance understanding of these athermal transient phenomena and provide an alternative to thermal annealing. Advancing understanding of ionization‐induced healing could accelerate its adoption in semiconductor technology for commercial devices. Herein, we investigate the ionization effects on the response of both defective and amorphous Ge using intermediate‐energy incident ions (12 MeV O).

## 2 Results: Structural and Defect Evolution

### 2.1 Crystalline Disorder and Damage Recover Revealed by Rutherford Backscattering Spectrometry in Channeling Geometry (RBS/C) Analysis

**Figure 1:**
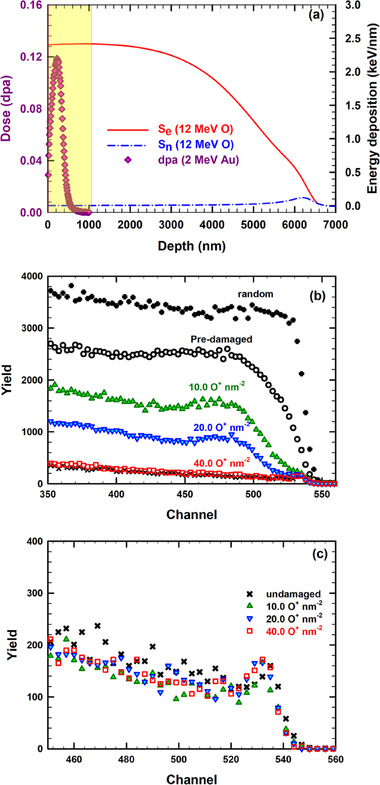
a) SRIM predicted electronic (*S*_e_) and nuclear (*S*_n_) energy loss for 12 MeV O ions in Ge along with predicted damage doses (dpa) for a fluence of 0.03 Au^−^ nm^−2^. Shaded area represents the surface region (≤ 1000 nm) of Ge single crystals characterized by RBS/C. b) The RBS/C spectra recorded for predamaged Ge with a maximum initial disorder fraction *f*_0_ ≈ 0.59 and sequentially irradiated at 300 K with 12 MeV O ions at the indicated fluences. c) The RBS/C for pristine Ge single crystals irradiated at 300 K with 12 MeV O ions at the indicated fluences.

**Figure** **1**a shows the electronic and nuclear energy loss calculated with the stopping and range of ions in matter (SRIM) code for 12 MeV O ions in Ge along with the predicted profile of damage dose (dpa) for a fluence of 0.03 Au^−^ nm^−2^. The evolution of the RBS/C spectra recorded along the <100> axis for the monocrystalline Ge predamaged with 2.0 MeV Au ions to an ion fluence of 0.03 ions nm^−2^ are shown in Figure 1b as a function of ion fluence for sequential irradiation with 12 MeV O ions at RT. For comparison, the RBS spectra recorded in random and channeling directions from a pristine crystal are also shown. The yields of the former and latter spectra represent the reference curve for random (amorphous) and pristine (undamaged) levels, respectively. Spectacularly, subsequent irradiation with 12 MeV O ions induces a dramatic decrease in ion channeling yield over the entire damage profile with increasing O ion fluence until the yield is indistinguishable from the corresponding aligned RBS/C yield for the unirradiated Ge. This demonstrates full recovery back to the virgin state for predamaged Ge following irradiation with 12 MeV O to an ion fluence of 40.0 ions nm^−2^ (see red open squares). In other words, full recovery of the ordered atomic structure, not previously reported in Ge, is observed at irradiation conditions as low as 12 MeV O ions.

**Figure 2:**
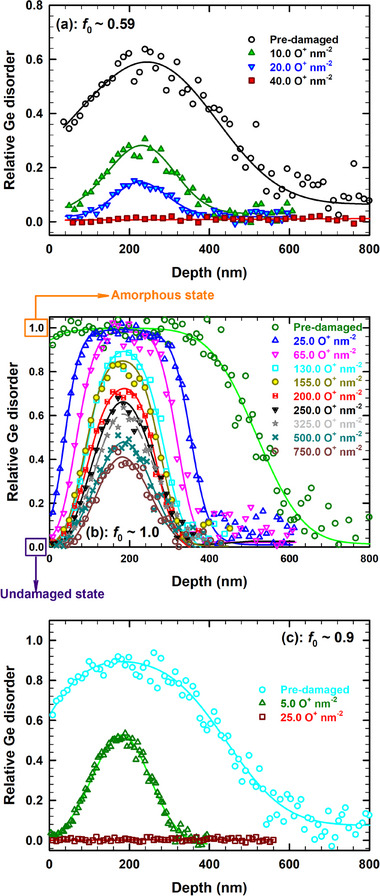
Evolution of relative Ge disorder profiles upon sequential irradiation at RT with 12 MeV O ions at the indicated fluences for: a) predamaged (*f*_0_ ≈ 0.59), b) preamorphized (*f*_0_ ≈ 1.00), and c) highly predamaged (*f*_0_ ≈ 0.9).

To better highlight this athermal recovery process, offline deconvolution analysis of each RBS/C spectra depicted in Figure 1b was performed utilizing an iterative approach^[^ ^29^ ^]^ to quantify the relative Ge disorder prior to and after sequential irradiation with 12 MeV O ions. The resulting disorder depth profiles are shown in **Figure** **2**a. In this analysis, Ge is amorphized if the magnitude of the relative disorder is equal to 1.0; whereas for the pristine crystal (undamaged), it is presumed to be 0. Irradiation with 2 MeV Au ions to an ion fluence of 0.03 ions nm^−2^ results in the creation of a damage profile with an initial maximum level of fractional disorder (*f*_0_) of ≈0.59 located at depth of about 240 nm (see open circles in Figure 2a), which is in good correlation with the maximum of the SRIM‐predicted dpa profile located at ≈220 nm (see pink filled diamonds in Figure 1a). According to previous studies, the crystalline‐to‐amorphous (c/a) phase transition in Ge irradiated at RT occurs in three steps,^[^ ^7^ ^]^ and the sample with *f*_0_ *≈* 0.59, corresponds approximately to the middle of step 2. It is predicted that damage morphology is likely governed by extended defects in step 2.^[^ ^7^ ^]^ For example, considerable damage recovery is observed after O irradiation at ion fluences up to 10.0 ions nm^−2^, as *f*_0_ decreases from 0.59 to 0.27. Notably, at an ion fluence of 40.0 O^−^ nm^−2^ (see red open squares in Figure 2a) the disorder level is very low over the entire predamaged layer (*f*_0_ *≈* 0.007). This very small amount of residual disorder suggests that a nearly damage‐free structure is obtained due to efficient athermal defect recovery. This behavior is similar to the behavior observed for predamaged SiC, where the ionization‐induced recovery led to a similarly low level of residual disorder that was attributed to the formation of small dislocation loops during damage recovery.^[^ ^27^ ^]^ The identification of residual defect structures present in the recrystallized region has yet to be determined; high‐angle annular dark field (HAADF) analysis on the predamaged Ge sample and subsequently irradiated with 12 MeV O ions to an ion fluence of 40.0 O^−^ nm^−2^ reveals some small variation in the atomic contrast (see Figure S2, Supporting Information) associated with the residual disorder following athermal recovery; here, we note that inelastic ionization‐induced recovery processes are often referred to as athermal because the transient inelastic thermal spikes and localized electronic excitations that lead to damage recovery, are only weakly dependent on the sample temperature during irradiation,^[^ ^31^ ^]^ in contrast to that reported for thermal annealing processes (see Section 3). However, it was observed that Ge samples are very sensitive to Gallium (Ga) focused ion beam processing, and further HAADF analysis could be taken into consideration to yield more accurate information on the nature of defect structures.

The recovery process in Ge observed in the current study is a natural consequence of the inelastic ionization processes,^[^ ^18^, ^27^ ^]^ since the *S*_e_ value of 12 MeV O ions remains approximately constant (2.4 keV nm^−1^) over the entire depth range studied (right axis, red solid line in Figure 1a); while in the same depth range, *S*_n_ for the same 12 MeV O ions (right axis, blue dashed‐dotted line in Figure 1a) is significantly lower, by a factor of ≈600, than the corresponding *S*_e_ value. Given the very low *S*_n_ value (≈0.004 keV nm^−1^) and high *S*_e_/*S*_n_ ratio (600), negligible damage creation from the nuclear energy deposition is expected within the RBS/C characterization region (i.e., 0 *< z <* 1000 nm). This assumption is confirmed by the RBS/C spectra for 12 MeV O irradiated pristine Ge (see Figure 1c) that do not show any increase in the backscattering yield compared with the ion channeling spectra recorded for unirradiated sample, within experimental errors, even at the highest ion fluences used in the current study (40.0 ions nm^−2^). This indicates that 12 MeV O ion irradiation alone did not induce any perceptible damage in the near surface region (≤1 µm) accessible to RBS/C. However, at the end of range for the 12 MeV O ions (and, hence, far from the predamaged layer), *S*_n_ dominates and creates defects that may evolve into an amorphous layer at high ion fluences, and such an amorphous region is expected to be found around the estimated O ion projected range shown in Figure 1a (not the focus of this current study). These findings reveal that competitive interactions between *S*_e_ and *S*_n_ occur (residual damage in this case is much smaller than the sum of separate *S*_e_ and *S*_n_ processes) because of the introduction of local disorder that sensitizes Ge to *S*_e_ effects and results in substantially reduced damage production (e.g., annealing) than that expected to occur from the sum of *S*_e_‐ and *S*_n_‐induced damage production processes alone.

Let us now focus on the ionization‐induced recovery efficiency for the case of preamorphized Ge, since high ion implantation doses are usually required that result in the creation of a buried amorphous layer. With this objective in mind, sequential irradiation of preamorphized Ge (*f*_0_ ≈ 1.0) with 12 MeV O ions has been performed. Note that the creation of an amorphous layer was the result of the 2.0 MeV Au ion irradiation at RT to an ion fluence of 0.1 ions nm^−2^. The resulting Ge disorder profiles prior to and after sequential irradiation with 12 MeV O ions at the indicated fluences are presented in Figure 2b following the analysis of the experimental ion channeling spectra (see Figure S1a, Supporting Information). Irradiation with 12 MeV O of preamorphized Ge initiates the recovery of the disorder occurring at the surface, at the buried c/a interface and at depths beyond the amorphous layer. Consequently, this process induces both recovery of defects beyond the amorphous layer and shrinkage of the amorphous layer thickness for increases in ion fluence, from 0 to 25 and 65 nm^−2^. Furthermore, the results in Figure 2b show that there is a transition from a decrease in the overall damage width to an onset of ionization‐induced healing over the entire damage profile as the ion fluence increases from 65 to 130 nm^−2^. This indicates that there exists an incubation fluence (*Φ*_0_) between 65 and 130 nm^−2^. This incubation fluence is due to time/fluence (e.g., amount of ionization energy deposited) required to recover fully amorphous material on both sides of the damage peak. At higher ion fluences, a further reduction in disorder over the entire damage profile is observed, and eventually the disorder level reaches a plateau. These results provide evidence that the energy transfer of only 2.4 keV nm^−1^ to electrons is an excellent external stimulus to induce epitaxial recrystallization of preamorphized Ge, from both the near‐surface and underlying c/a interface under subsequent 12 MeV O ion irradiation at RT. The occurrence of recrystallization at the buried amorphous‐crystalline (a‐c) interface is not surprising because the defects present in both surrounding defective regions and at the c/a interface fuels this process; on the other hand, one should consider that the a‐Ge is not fully amorphous on or near the surface because otherwise epitaxial crystallization from the near‐surface is impossible in the absence of tiny crystalline seeds that are used as “remanent memory” to recover the initial crystallographic direction (i.e., <100>). This leads to an interesting question, how can the incubation fluence be determined? This is discussed in the 3.1 subsection.

Moreover, the comparative analysis of the ionization‐induced recovery efficiency between partially damaged and preamorphized Ge (complete vs incomplete recovery) reveals another unexpected outcome, namely that there exists a dependence of complete recovery on how the particular damage state is created. To confirm our hypothesis that different recovery processes are dependent on how the damage state is created, we designed another independent experiment. In this case, a predamaged disorder level of 0.9 was created in pristine Ge by irradiation with 2.0 MeV Au ions at RT to an ion fluence of 0.05 ions nm^−2^. This predamaged Ge (*f*_0_ ≈ 0.9) sample was subsequently irradiated with 12 MeV O ions to investigate athermal recovery kinetics as a function of fluence, as shown in Figure 2c and to compare to the recovery of the residual damage state, also with *f*_0_ ≈ 0.9, created in preamorphized Ge following irradiation with 130 O^+^ nm^−2^. The results demonstrate that this initial pre‐existing disorder level decreases with increasing ion fluence and nearly complete recover is observed at a fluence of 25.0 ions nm^−2^ (residual *f*_0_ ≈ 0.008); whereas for the same disorder level (*f*_0_ ≈ 0.9) formed upon recovery of preamorphized Ge, the recovery is minor (less than a few percent) as the fluence is increased by 25 ions nm^−2^ from 130 to 155 ions nm^−2^ (see Figure 2b). This rapid recovery in predamaged Ge (*f*_0_ ≈ 0.9) is in stark contrast to that observed for the same disorder level (*f*_0_ ≈ 0.9) created upon recovery of preamorphized Ge (see Figure 2b) and presents a clear indication for the occurrence of completely distinct damage recovery mechanisms due to differences in the how the structure of the defective layers are created (disorder created in a pristine crystal versus residual damage upon ionization‐induced recovery). This means that the efficiency of ionizing irradiation for damage removal (and hence, the recovery cross‐sections) in Ge substantially depends on the type and concentration of the damage produced, which confirms our hypothesis.

### 2.2 Phase Stability and Strain Evolution from X‐Ray Diffraction (XRD) Analysis

XRD is extremely sensitive to atomic displacements u(z) which, for the h00 reflections, can be decomposed as follows

(1) u(z)=e(z)×z+δu(z)

where *e*(*z*) is the strain distribution as a function of depth *z* (i.e., nonrandom displacements), and *δu*(*z*) correspond to random displacements. These two components affect the XRD signal in a very different fashion. Whereas strain produces a shift of the peak, here responsible for the extension of the signal toward the lower angle region, the disorder modulates the diffracted intensity via the so‐called static‐Debye‐Waller (DW) factor

(2) $$SW\left(z\right)=\langle \exp\left[iQ\delta u(z)\right]\rangle$$

where *Q* is the length of the scattering vector (4 π sin*θ*/*λ*) and the average < > is taken over the statistical distribution of the displacement at depth *z*. The DW factor quantifies the level of disorder induced by irradiation. High level of disorder leads the exponential to vanish, i.e., DW = 0. On the contrary, for a perfect crystal, there are no random displacement so that DW = 1. In short, the DW factor can be viewed as the volume fraction of nondisordered material for a given hkl reflection. RaDMaX‐online allows to determine both the strain and the DW profiles via a simulation of the XRD data.

**Figure 3:**
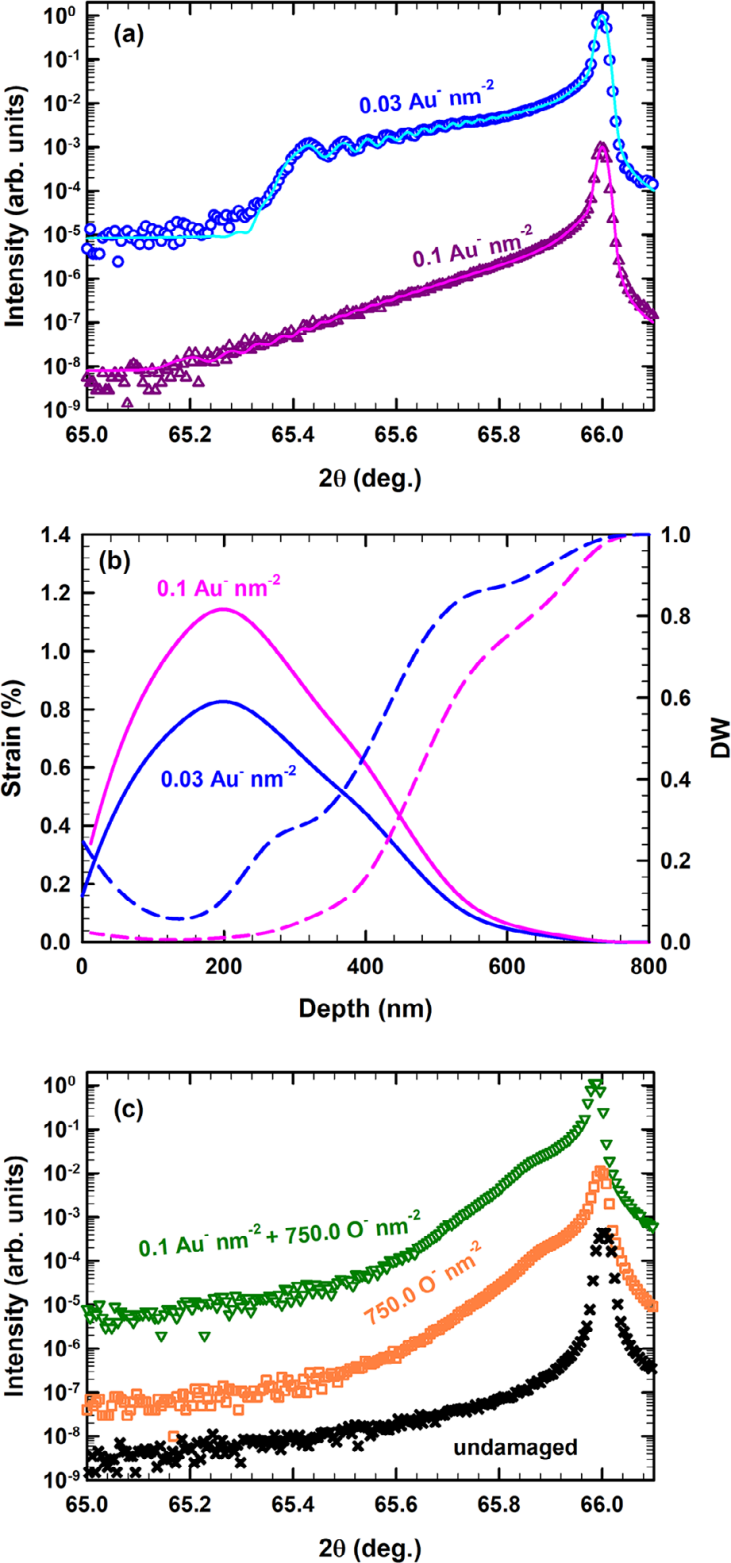
a) *θ*‐2*θ* scans across the 004 Bragg reflection of Ge (symbols) irradiated with 2 MeV Au ions at indicated ion fluences. Solid lines are best fits with RaDMaX‐online^[^ ^30^ ^]^ in order to extract the strain and disorder depth profiles (DW) in the irradiated region. The corresponding strain (solid lines) and DW (dash lines) profiles are illustrated in (b). The *θ*‐2*θ* scans before and after irradiation with 12 MeV O ions at the indicated ion fluences of both pristine and predamaged Ge samples are shown in (c). The curves are shifted vertically for clarity.

In **Figure** **3** we display the *θ*‐2*θ* experimental scans recorded across the 400 Bragg reflection of Ge (symbols) irradiated with 2 MeV Au ions at ion fluences of 0.03 and 0.1 ions nm^−2^. Apart from the intense narrow peak on the high‐angle side (≈66°) emanating from the unirradiated part of Ge, an additional signal is observed on the low‐angle side for both samples. This signal indicates the presence of a tensile strain distribution normal to the surface, consecutive to Au irradiation. For the low fluence (0.03 ions nm^−2^), a fringe pattern is clearly visible from which the strain and disorder depth profile can be retrieved with RaDMaX‐online.^[^ ^30^ ^]^ For the highest Au fluence (0.1 ions nm^−2^), the signal from the damage region further shifts toward lower angles, and more importantly, the fringe pattern vanishes, which indicates a strong increase in random atomic displacements, i.e., disorder. Although no fringes are clearly visible, the strain and disorder profile can still be retrieved by extrapolating from the lower fluence. The evolution upon increasing fluence can be ascribed to either a highly disordered Ge crystalline structure in the irradiated region, or to an amorphized material, as revealed by ion channeling. The simulated curves are superimposed with the experimental data in Figure 3a (solid lines). It is worth noting that very good agreement is obtained between simulated and experimental data. The strain and disorder profiles resulting from the simulations are shown in Figure 3b.

Let us first discuss the strain distributions (continuous lines). The strained region spans a depth of ≈800 nm, and a maximum of strain, 0.82%, is observed at ≈ 200 nm. These observations are in excellent agreement with the results obtained by RBS/C and predicted by SRIM. Upon increasing fluence, the strain increases; we will discuss this trend in more detail with regards to the evolution of the disorder distribution. For the sample irradiated at 0.03 ions nm^−2^ the disorder reaches values as low as 0.06, yet it remains crystalline as indicated by the presence of interference fringes in the XRD data. For the higher fluence, the DW factor decreases to a value of 0.0008 at its minimum. Generally, an empirical observation is that DW values below 0.05 can be considered as fully disordered or amorphous. In this case, this corresponds to the region between the surface and ≈300 nm below the surface. Since lattice strain is undefined for an amorphous material, the highest level of strain measured in the crystalline region is 0.92%, at 300 nm below the surface.

XRD data recorded upon sequential irradiation at RT with 12 MeV O ions to an ion fluence of 750.0 ions nm^−2^ (≈2.5 dpa at the Au‐induced damage peak) of both pristine and preamorphized (*f*_0_ ≈ 1.00) samples are shown in Figure 3c. For comparison purposes, the XRD data from the undamaged sample are also shown. In the present case, the lack of significant features in the XRD data, and the lack of a sample with visible features in the XRD curves from which to extrapolate, prevents any reliable simulation to be performed. Nonetheless, a qualitative description of the curves still provides meaningful results. Let us first consider the case of Ge irradiated solely with O ions. A secondary broad peak can be detected around 65.88°, which corresponds to a strain level of 0.17%. Electronic energy loss does not usually give rise to lattice strain. Hence, the most likely reason for the existence of tensile strain from O irradiation is the nuclear energy loss appearing at the ions end of range (> 6 µm). Regarding the case of the preamorphized sample that was subsequently irradiated with 12 MeV O ions to an ion fluence of 750.0 ions nm^−2^ (or ≈2.5 dpa at the Au‐induced damage peak), the most striking feature is that it is identical to the case of the Ge crystal solely irradiated with O ions. In other words, any signal coming from Au irradiation vanished, which points to a full relaxation of the Au induced strain.

### 2.3 Atomic‐Level Damage Mechanisms and Defect Recovery Pathways from Molecular Dynamics (MD) Simulations

**Figure 4:**
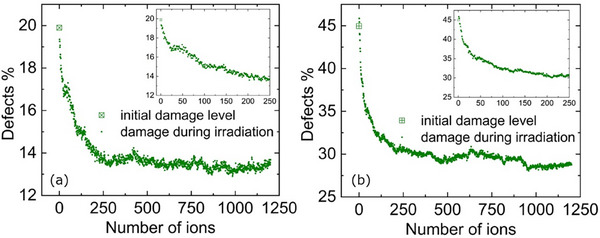
MD simulations of the evolution of defect levels in Ge with increasing number of 12 MeV O thermal spikes in two systems with different initial levels of pre‐existing defects: a) 20% and b) 45% defects. The insets show the damage for the first 250 ions.

In **Figure** **4** we show the evolution of the damage level in the two predamaged systems for 1200 ions, obtained from the molecular dynamics (MD) simulations of 12 MeV O irradiation (i.e., thermal spikes). The initial level of pre‐existing defects is noted. The defect level decreases 6.3% (drops from about 20% to 13.7%) for the first system shown in (a) and 16.3% (drops from about 45% to 28.7%) in the second system shown in (b). In other words, in the system with lower pre‐existing defect concentration, the recovery was 31.5%, while in the system with higher concentration, the recovery was 36.2%. These results also show that a higher pre‐existing damage level leads to greater recovery of defects. For the system with the higher disorder, a few more defects are formed for the first few ions, which are negligible and may be due to statistical uncertainty in identifying defects; however, the high concentration of defects in both cases results in defect recombination processes. As shown in the insets, a faster recovery rate is observed at the beginning of the simulated irradiation, for about the first 50 ions, and a slower recovery rate is observed with increasing number of overlapping ions (or fluence), from 250 to 500 ions. Eventually, the defect level reaches a plateau as the number of overlapping ions increases to 1200 ions. In other words, the results illustrated in Figure 4 shows an exponential drop‐to‐saturation dependence. We note that while there is no direct correspondence of the level of defects to the disorder measured experimentally, we capture the effects of annealing at mid and high disorder levels.

**Figure 5:**
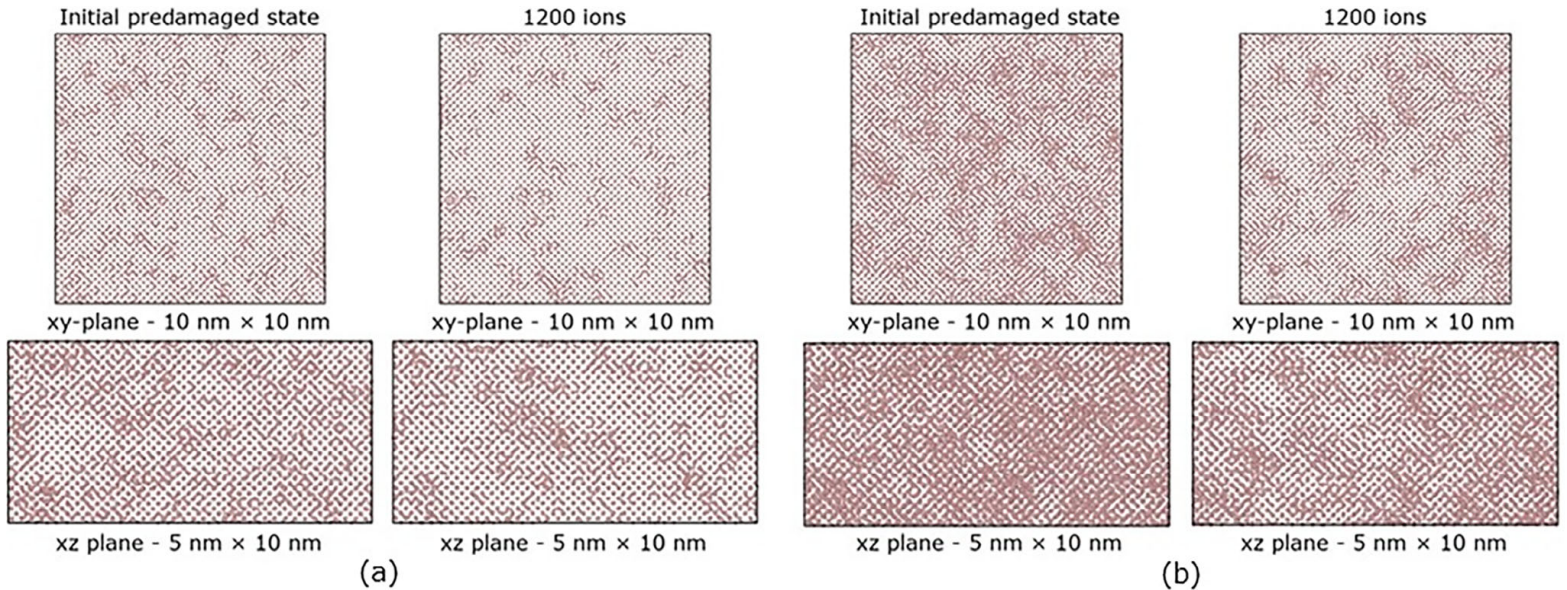
Initial predamaged structure and structure after simulation of irradiation with 1200 12 MeV O ions for two systems with different pre‐exiting defect level, a) 20% defects and b) 45% defects. The top rows show the top view of the MD box (*xy*‐plane) and the bottoms rows show the side view (*xz*‐plane) of the MD box.

In **Figure** **5**, we show the initial (predamaged) and final (after irradiation with 1200 12 MeV O ions) structures for the two systems. The top row shows the top view of the MD box (*xy*‐plane) and the bottom row shows the side view of the MD box (*xz*‐plane), before and after irradiation. Here, the effects of the irradiation on the structure can be observed, where clusters and defects at pockets of damage recombine, and the pockets become visibly smaller or disappear, revealing crystalline areas. The recovery of defects in defect pockets, as well as isolated point defect recombination, can also be seen in the movies provided in the supplementary material. The higher concentration of defects in the defect pockets enables faster recombination; as the surviving (available) defects are continuously consumed by the recovery process, the recombination rate is progressively reduced. More specifically, the recombination rate decreases as the defect pockets are consumed, and the recovery process tends to level off at increasing number of overlapping ions. Furthermore, the results unambiguously show an increase in the level of residual disorder when the initial level of pre‐existing defects is higher (20% vs 45% defects). In other words, the initial level and types of pre‐existing defects have a strong effect on the level and nature of the residual disorder. The residual defects/strain or interfacial dynamics (or both) may be considered as a limiting factor of the observed differences, which may explain reasonably the observed incomplete recovery in the case of preamorphized Ge.

## 3 Discussion

### 3.1 Ionization‐Induced Recovery and Strain Relaxation in Ge at RT

It is well‐established that electronic energy dissipation induces a highly localized thermal spike along the ion path, and the associated transient processes occur on ps to ns time scales.^[^ ^31^ ^]^ Initially, the system undergoes rapid quenching on ps time scales (stage 1), before thermal equilibrium is achieved on ns time scales (stage 2). Between stages 1 and 2, defect and atomic mobility can be enhanced and, thus, promote athermal recovery of pre‐existing collisional defects and dynamic annealing of defect production (point defects) along the ion path. The entire process is often considered as “athermal transient phenomena” due to its weak or nonsubstantial dependence on temperature, which is opposite to what one usually would expect for thermally activated processes.^[^ ^31^ ^]^ Consequently, this process is responsible for the defect healing and strain relaxation observed in Ge at RT. Athermal ionization‐induced recovery has been also observed in predamaged Ge as a reduction in disorder at the damage peak from ≈0.5 to ≈0.3 under 100 MeV Ag ion irradiation at RT to an ion fluence of 2.0 ions nm^−2^;^[^ ^1^ ^]^ the corresponding *S*_e_ value within the predamaged layer is ≈16.35 keV nm^−1^ as compared to only 2.4 keV nm^−1^ for 12 MeV O ions. The previous independent transmission electron microscopy (TEM) analysis associated with that work^[^ ^1^ ^]^ demonstrated that the initial damage morphology of the sample with *f*_0_ *≈* 0.5 (similar to that in the current work) is dominated by damaged pockets within the crystalline surroundings,^[^ ^1^ ^]^ and thus the morphology of the damage structure in our study (*f*_0_ ≈ 0.59) is expected to be similar. Our current MD simulations results (see above the results of MD simulations) support the assumption that mainly damaged pockets within the crystalline surroundings are expected to be found for the sample with *f*_0_ ≈ 0.59. In related work, it was also revealed that the pre‐existing damage (*f*_0_ ≈ 0.8) was almost fully healed and the ordered atomic structure was confirmed under irradiation at 475 K with 100 MeV Ag ions to an ion fluence of 1.0 ions nm^−2^.^[^ ^2^ ^]^ However, this process is no longer a completely athermal transient phenomenon, since additional thermal energy is present during irradiation, which may be detrimental if the final objective is to fully restore the atomic structure without affecting the uniform in‐depth distribution of dopants. Here, we note that according to SRIM calculation the maximum atomic Au concentration does not exceed 0.001 at% for the highest Au fluence used in this study (0.1 Au^+^nm^−2^); such a small impurity content is below the detection limit of the applied method and thus, we cannot monitor the migration of the preimplanted Au ions in Ge before and after ionizing irradiation. Although such studies are pivotal for advancing prospects for application of ionization‐induced recovery process in the fabrication of semiconductors, to our knowledge, there is only one study where the influence of ionizing irradiations on the migration of preimplanted Ag ions in SiC has been studied by means of RBS.^[^ ^32^ ^]^ Their experimental findings do not exhibit any perceptible difference in Ag yield, beyond the uncertainty of the experimental measurements, indicating that SHI irradiation did not promote any significant migration/redistribution or loss of Ag at concentrations accessible to RBS.^[^ ^32^ ^]^ While no visible Au signal is detected in the RBS spectra in the current study, a weak or negligible migration of preimplanted Au may be expected upon O irradiation in analogy with the experimental findings of Abdalla et al.^[^ ^32^ ^]^

Now, let us return to the results of Hooda et al.^[^ ^1^, ^2^ ^]^ that also revealed that irradiation of amorphous Ge under similar conditions results in volume expansion and nanorod formation within the amorphous layer instead of recrystallization. Here we note that ion track formation represents the embryonic precursors to the porous structure formation in amorphous Ge by SHI irradiation.^[^ ^16^ ^]^ For 100 MeV Ag, the *S*_e_ is ≈16.35 keV nm^−1^ within the amorphous layer, which is above the threshold *S*_e_ value (*S*_e_^th^) for tracks and void creation in amorphous Ge under SHI irradiations reported in literature, e.g., *S*_e_^th^ > 10.0 keV nm^−1^.^[^ ^33^ ^]^ Thus, the SHIBIEC process studies by Hooda et al.^[^ ^1^, ^2^ ^]^ was restricted to only predamaged disordered states below the fully amorphous state. It might be that the reason for that lies within the occurrence of a significant reduction in the *S*_e_^th^ for synergistic effect (damaging) with increasing amount of pre‐existing damage (partially damaged vs preamorphized), as it was observed in KTaO_3_.^[^ ^34^ ^]^ In other words, both synergistic (enhances the previous damage) or competitive (decreases the previous damage) effects can coexist, in the same material for the same *S*_e_ value either above or below an initial disorder level threshold, respectively, demonstrating complex interactions between *S*_e_ and pre‐existing atomic defects. This drawback somehow limits the application of SHIBIEC process in the fabrication of Ge‐based devices, since high ion implantations doses are usually required, resulting in the creation of an amorphous layer. The recrystallization of preamorphized Ge was previously observed only under ex‐situ thermal treatments at elevated temperature^[^ ^2^, ^7^ ^]^ that is exclusively related to thermally driven annealing; whereas the recovery process observed in the present study arises as a result of athermal transient generated phenomena. Since for 12 MeV O ions the *S*_e_ value (2.4 keV nm^−1^) is far below the *S*_e_^th^ for track formation in Ge (≈10.0 keV nm^−1^), the creation of ion tracks is hindered; this low value explains why athermal ionization‐induced recovery is not restricted to only predamaged state, as in the case of 100 MeV Ag ions. In other words, these findings clearly demonstrate that the a/c phase transition in Ge is reversed under 12 MeV O irradiation (current study), which contrasts with that observed previously under SHI irradiation.^[^ ^2^, ^7^ ^]^ In the case of SHI, instead of annealing, the opposite effect of damage production (formation of ion tracks) is observed, and recrystallization of amorphous Ge was not observed. This indicates that the temperature transient along the 12 MeV O path is insufficient to trigger local melt quenching (ion track formation) based on the *S*_e_^th^ for track formation in Ge determined previously,^[^ ^33^ ^]^ but is high enough to promote defect recovery in Ge by enhancing defect mobility via thermal spikes, as shown in the MD simulations.

**Figure 6:**
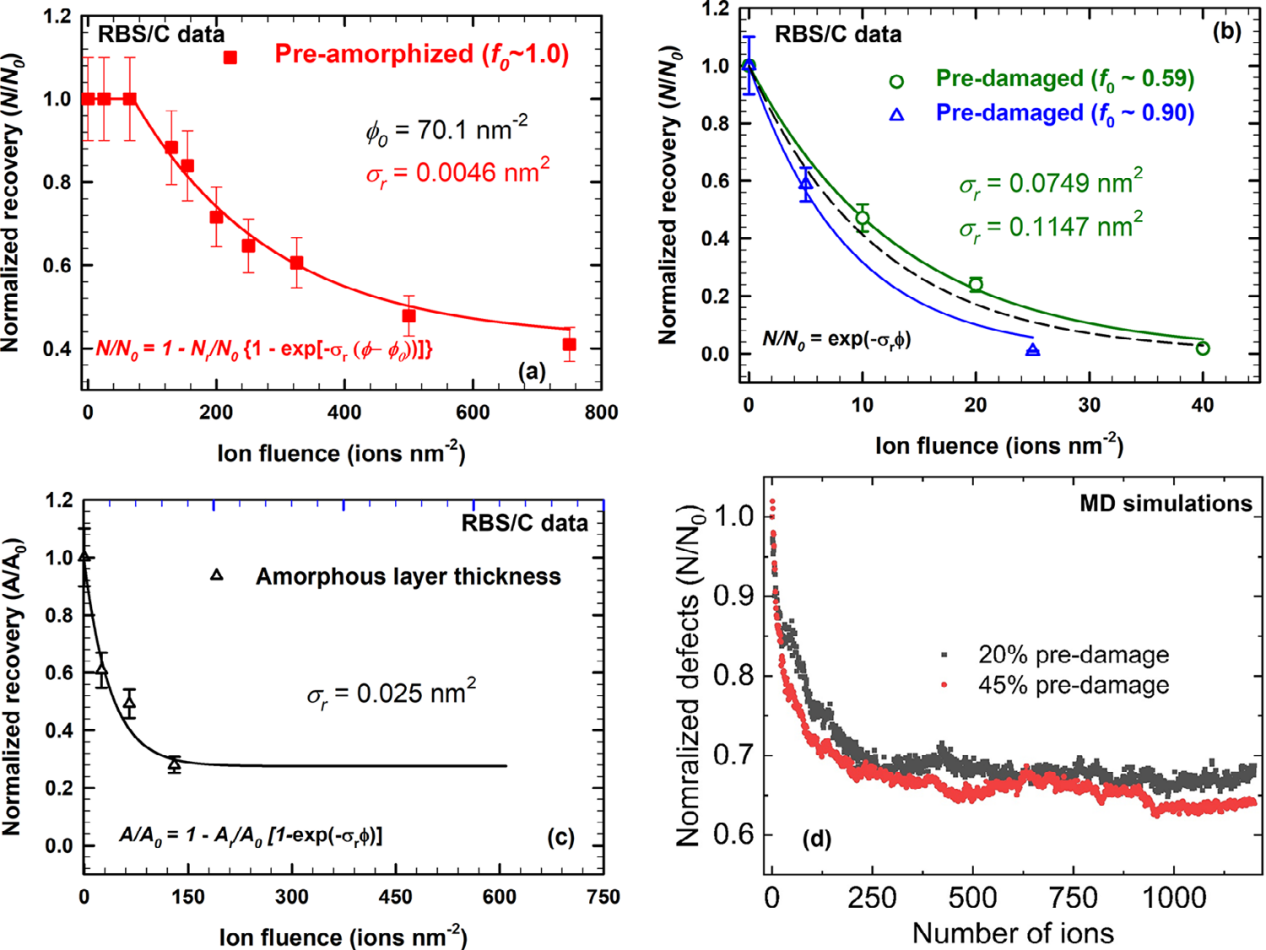
Evolution of the normalized recovery at the damage peak (*N*/*N*_0_) as a function of 12 MeV O fluence, obtained from RBS/C analysis: a) in preamorphized and b) in the predamaged Ge, respectively. In both cases, lines are best fits with Equations (3) and (5) for fully amorphous and predamaged Ge, respectively. Additionally, a single fit of Equation (5) to both recovery data sets is also superimposed, (b) as a dash black line. c) Evolution as a function of 12 MeV O fluence of the amorphous layer thickness recovery efficiency. d) normalized recovery from MD simulations of thermal spike events for 12 MeV O ions in Ge with different initial levels of pre‐existing defects (i.e., 20% vs 45% defects) as a function of number of ions.

The evolution of the experimental disorder curves shown in Figure 2b reveals the existence of an incubation fluence between 65 and 130 O nm^−2^ before measurable recovery at the damage peak occurs. Essentially, before reaching this incubation fluence, the recovery process is related to ionization‐induced recrystallization and defect‐annihilation at the c/a interface and defect recombination at depths beyond the c/a interface;^[^ ^24^ ^]^ however, above this incubation fluence, recovery occurs over the entire remaining damage thickness but more slowly with increasing fluence without achieving the pristine state (residual damage saturates). The existence of this incubation fluence before the onset of recovery over the entire remaining damage thickness is because the thick amorphous layer is consumed by epitaxial recovery process until a continuous amorphous layer no longer exists, at which point sluggish recovery processes of a complex residual damage structure occur over the entire remaining damaged thickness, which exhibits a Gaussian profile. Interestingly, the width (thickness) of these Gaussian damage profiles does not change much with increasing fluence, indicating similar damage structures and recovery processes over the remaining damage thickness. In order to determine the value of this incubation fluence, the normalized recovery of relative Ge disorder at the damage peak (*N*/*N*_0_) as a function of 12 MeV O ion fluence (*Φ*) is obtained from the RBS/C analysis and plotted in **Figure** **6**a, for preamorphized Ge (see disorder profiles illustrated in Figure 2b). Above the incubation fluence, this plot clearly depicts the same trend as reported previously in other predamaged crystalline materials (e.g., SiC,^[^ ^27^ ^]^ KTaO_3_,^[^ ^35^, ^36^ ^]^ and Si^[^ ^18^ ^]^) irradiated with intermediate‐energy ions, where the damage recovery exhibits a simply exponential decay dependence on ion fluence (or number of overlapping ions). The following formula has been adapted to model this dependence and to determine both incubation fluence (*Φ*_0_) and recovery cross‐section (*σ*_r_)

(3) $$N/N_{0}=1-N_{r}/N_{0}\times \left\{1-\exp\left[-\sigma_{r}\times \left(\Phi -\Phi_{0}\right)\right]\right\}$$

where *N*_r_/*N*_0_ represents the recoverable fraction of disorder at the damage peak. A fit of [Equation (3)] to the RBS/C data is shown in Figure 6a as a solid line. Note that the model curve fit demonstrates a very good representation of the experimental data. This demonstrates that the model is capable of determining incubation fluence, recovery cross‐section and recoverable fraction of disorder. This yields an incubation fluence of 70.0 ± 7.0 nm^−2^ that is needed prior to the onset of recovery at the damage peak over the remaining damage thickness and is no longer limited to only the c/a interface. The other derived fitting parameter (*σ*_r_) is shown in **Table** **1**. The recovery kinetics for preamorphized Ge will be discussed in the following subsection taking into account the *Φ*_0_ as a key parameter.

**Table 1:** Athermal ionization‐induced recovery cross section, *σ*_r_, determined at the damage peak for different predamaged levels in Ge induced upon irradiation with 12 MeV O ions. The recoverable fraction for each *f*_0_ is also included. If one assumes that recrystallization occurs inside an ion track that is cylindrical, the corresponding average cylinder diameter (*d*) is also included.

Initial disorder levels	Recoverable fraction	*σ*_r_ [nm^2^]	Eq.	*d* [nm]
*f*_0_ *≈* 0.59	1	0.0749 ± 0.02	(5)	0.30 ± 0.1
*f*_0_ *≈* 0.90	1	0.1147 ± 0.03	(5)	0.38 ± 0.1
*f*_0_ *≈* 1.0	0.63	0.0046± 0.001	(3)	0.072 ± 0.01

To our knowledge, this remarkable reversal of the crystalline‐to‐amorphous phase transformation in Ge, which occurs at RT, has not been previously reported. Somewhat equivalent recovery processes of fully amorphized crystals have also been reported for Ge^[^ ^24^ ^]^ but related, in all cases, to dominant high nuclear energy loss in preamorphized Ge, and operative only at irradiation temperatures above RT, unlike in the present study. Moreover, the experimental data available up to now have indicated that ionization‐induced recrystallization at RT in preamorphized semiconductor materials (i.e., SiC^[^ ^37^ ^]^ and Si^[^ ^28^ ^]^) only reduces the thickness of the amorphous layer, while this is not the case in the present study; this may be related to insufficient transient thermal budgets from the “thermal spike” (not enough energy to entirely consume the amorphous layer). However, it was also argued that a higher resolution TEM analysis would be necessary before a more precise conclusion could be made. Nevertheless, this led the authors^[^ ^37^ ^]^ to claim that the presence of crystalline seeds can further mediate recrystallization process over the entire damage thickness and not be limited to only the c/a interface (shrinkage of the amorphous layer). Here, it is also worth noting that previous studies have not used such high irradiation fluences as in the present case for Ge. Especially in the case of swift heavy ions, it is impossible to reach such high fluences within a reasonable irradiation time. Nevertheless, it was found that the SHIBIEC process studies by Hooda et al.^[^ ^1^, ^2^ ^]^ was restricted to only predamaged disordered states below the fully amorphous state, in contrast to that reported in this study.

For *Φ* < *Φ*_0_ the recovery appears as a combination of ionization‐induced (or thermal spike induced) defect recombination in the defective crystal structure (i.e., defects beyond the c/a interface) and ionization‐enhanced recrystallization at the c/a interface due to ionization‐enhanced or thermal spike enhanced defect mobility at or near the interface. Usually, the damage location and the thickness of the fully amorphous layer estimated by RBS/C matches the depth distribution of defects measured by TEM, if the depth scale for the damage profile is corrected for swelling (density decrease in the amorphous structure). For example, an average of 15% volume swelling for depth scale damage profile correction has been applied in amorphous SiC.^[^ ^37^ ^]^ Even though data at 70.0 nm^−2^ are absent, but exists for 130 nm^−2^, one should expect that once the incubation fluence of 70.0 nm^−2^ is reached, the disorder profile becomes somewhat Gaussian and is no longer fully amorphous at the damage peak. For *Φ* ≥ *Φ*_0_, the recovery of the amorphous/damage thickness is no longer advancing significantly or reaches a plateau, i.e., the full width at half maximum of the disorder profiles does not decrease appreciably with increasing O fluence (see Figure 2b). Since this region was once fully amorphous, we can only assume that the residual disorder in this region is primarily some residual range of complex defect structures that cannot readily recrystallize due to insufficient transient thermal budgets from the “thermal spike” (not enough energy to fully eliminate). Although we do not know the microstructure corresponding to the transition at the incubation fluence (determined by fit), it should certainly not be the same as the disorder profile created in a pristine crystal by the accumulation of irradiation damage, which would consist mostly of point defects and amorphous clusters. However, the damage state in the case of *Φ* ≥ *Φ*_0_, mostly likely consists of randomly distributed amorphous clusters that recover by recrystallization at the now 3D c/a interface, which is slower without the presence of dense concentrations of point defects and cluster. Indeed, for *Φ* ≥ *Φ*_0_, the recovery is measurably slower than that for *Φ* < *Φ*_0_ because the defects present in both surrounding defective regions, as it was also observed in our MD simulation, and at the c/a interface are mostly consumed before *Φ*_0_ is reached. Consequently, the recrystallization process for *Φ* ≥ *Φ*_0_ becomes much slower, or less efficient, because the residual random distribution of amorphous domains/clusters lacks sufficient interfacial and surrounding defect structures to drive the recrystallization process at the same kinetics as for *Φ* < *Φ*_0_.^[^ ^37^ ^]^ The occurrence of random recrystallization (recrystallization along different directions) is totally excluded, since the formation of a polycrystalline nanostructure will result in a high backscattering yield that is not aligned with the original single crystal (not the case in the present study). Based on the ion channeling data provided above, we hypothesize that the presence of point defects will mediate the near full recovery of a pre‐existing, but different, damage state at the same disorder level (*f*_0_ ≈ 0.9) that is created in a pristine crystal by the accumulation of irradiation damage up to the desired disorder level (not a residual disorder state created upon annealing of a buried amorphous layer). The recovery is expected to be like the one observed for the sample with an initial disorder level of 0.59 shown in Figure 2a. Hence, in order to confirm this hypothesis, we designed another independent experiment (see next subsection).

Furthermore, the XRD curve recorded on the preamorphized sample and subsequently irradiated with 12 MeV O ions show a significant strain relaxation effect (Figure 3c), during which the initial strain generated by Au irradiation undergoes partial relaxation due to both the annealing and defect reorganization processes, as it is known that *S*_e_ relaxes the pre‐existing defect structure.^[^ ^31^, ^38^ ^]^ A colossal level of strain relaxation is expected in predamaged Ge with a maximum initial disorder fraction *f*_0_ ≈ 0.59 and sequentially irradiated at 300 K with 12 MeV O ions, as the overall damage fraction drops off with increasing fluence and approaches zero. Further additional XRD analysis will be necessary to verify this, especially at intermediate O fluence where no full relaxation is expected (and hence, measurable strain). This study demonstrates that *S*_e_ from MeV ion irradiation, even at a very moderate level of ≈2.4 keV nm^−1^, has a significant impact on pre‐existing damage, showing that electron–phonon coupling induced defect healing in Ge is inherently connected with the strain relaxation.

### 3.2 Initial Damage Level Disorder‐ and Defect Nature‐Dependent Recovery Cross‐Section

To provide further insights into the observed athermal ionization‐induced recovery kinetics and consequently derive the recovery cross section (*σ*_r_) associated with these processes, the normalized recovery is plotted as a function of 12 MeV O ion fluence (Φ), as shown in Figure 6b, by considering *N*/*N*_0_ of disorder profiles shown in Figure 2a,c. Consistent with previous studies, the following equation is used to model ionization‐induced recovery and to extract *σ*_r_

(4) $$N/N_{0}=1-N_{r}/N_{0}\times [1-\exp\left(-\sigma_{r}\times \Phi )\right]$$

In the absence of an incubation fluence (*Φ*_0_ = 0), Equation (3) reduces to Equation (4). If there is full recovery of the ordered atomic structure,^[^ ^39^ ^]^ *N*_r_/*N*_0_ becomes equal to 1; hence, *σ*_r_ given by Equation (4) is simplified to

(5) $$N/N_{0}=\exp\left(-\sigma_{r}\times \Phi \right)$$

Since in the predamaged samples with *f*_0_ *≈* 0.59 and ≈0.90, the residual damage at the highest fluences in this study is very low over the entire predamaged layer (i.e., *f*_0_ *≈* 0.007), we assume that full recovery occurs at higher fluences. A fit for each predamage level is shown as solid green and blue lines in Figure 6b for predamaged samples with *f*_0_ *≈* 0.59 and ≈0.90, respectively. These fits yield the recovery cross‐sections that are given in Table 1. Additionally, a single fit of Equation (5) to both recovery data sets is also superimposed Figure 6a as a dash black line. The values of *σ*_r_ obtained from these fits demonstrate that this competitive (recovery) process in nonamorphous Ge exhibits a strong dependence on the level of pre‐existing disorder, since the values of *σ*_r_ increases with the amount of initial pre‐existing damage. Specifically, the values of *σ*_r_ increase from 0.0749 ± 0.01 nm^2^ for a pre‐existing fractional disorder of 0.59 to 0.1147 ± 0.01 nm^2^ for a pre‐existing fractional disorder level of 0.90. These findings clearly show that recrystallization is faster in the sample with a higher pre‐existing fractional disorder level of 0.90 than in the sample with lower pre‐existing (*f*_0_ *≈* 0.59), which is consistent with our MD results. While separate fits were performed for each data set, which yielded the above‐mentioned values of *σ*_r_, but with high uncertainty (i.e., ≈30%) due to limited data,^[^ ^35^, ^37^ ^]^ a single fit of Equation (6) to both recovery data sets was also applied, which yielded a recovery cross section of 0.082 ± 0.01 nm^2^.

As discussed above, shrinkage of the buried amorphous/damaged layer thickness is observed for *Φ* < *Φ*_0_ ions in the preamorphized sample (Figure 2b) and since the representation of *N*/*N*_0_ versus ion fluence cannot be used to capture recovery kinetics in this case (*f*_0_ is always equal to ≈ 1), the relative areal density under amorphous layer (*A*/*A*_0_) is plotted against ion fluence in Figure 6c. This plot clearly depicts the same trends as reported previously in other predamaged crystalline materials (e.g., SiC,^[^ ^27^ ^]^ KTaO_3_,^[^ ^35^, ^36^ ^]^ and Si^[^ ^18^ ^]^) irradiated with intermediate‐energy ions, where the fluence (or number of overlapping ions) dependence of damage recovery can also be parameterized by a simple exponential decay function

(6) $$A/A_{0}=1-A_{r}/A_{0}\times \left[1-\exp\left(-\sigma_{r}\times \Phi \right)\right]$$

where *A*_r_/*A*_0_ represents the recoverable fraction of the areal amorphous density (or recoverable fraction of amorphous layer thickness) and Φ is the ion fluence. In Figure 6c, only the data from 25.0 to 130.0 ions nm^−2^ are used. For this case (fully amorphous layer), the value of *σ*_r_ is found to be 0.025 ± 0.01 nm^2^; whereas the fit for *Φ* ≥ *Φ*_0_ (not shown here), yields a much lower value: 0.009 ± 0.002 nm^2^. These findings clearly show that recrystallization is faster in the fully amorphous/damaged layer (for *Φ* < *Φ*_0_) than in partially amorphous crystal (for *Φ* ≥ *Φ*_0_). This finding can be ascribed to the presence of defective crystalline regions surrounding the amorphous layer that enhance recovery kinetics (for *Φ* < *Φ*_0_), but they are continuously consumed during the recrystallization/recovery of amorphous/damaged layer. Thus, it is expected that the decrease in the presence of mobile local defects will slow kinetics (for *Φ* ≥ *Φ*_0_). Normalized recovery due to MD thermal spikes from 12 MeV O ions is shown in Figure 6d for Ge with different initial levels of pre‐existing defects (i.e., 20% vs 45% defects) as a function of number of ions. These plots show an exponential drop‐to‐saturation dependence. The MD results are in reasonable agreement with the RBS/C data, also shown, which confirms defect recovery is driven by ionization‐induced thermal spikes from 12 MeV O ions. The differences between ion channeling and MD results (full vs incomplete recovery) may be related to fact that the simulations do not allow for recombination processes that occur on longer time scales in the experiments. Additionally, the irradiated volume in the MD simulations is limited and subjected to ions passing through the same path, unlike in experiment where overlapping energy depositions from different ion paths contribute to the defect recovery.

Before highlighting the novelty of the present study compared to published work in this subject area, we would note that there is very limited data on ionization‐induced recovery in Ge that can be used to ascertain whether comparable or different recovery mechanisms are at work in a‐Ge and a‐SiC or a‐Si. However, it is believed that a combination of material‐specific damage formation and ionization‐induced recovery processes may be operative. More specifically, recovery occurs over the entire damaged thickness only in partially amorphous SiC samples;^[^ ^37^ ^]^ while recrystallization is restricted at the buried amorphous‐crystalline (a‐c) interface in a‐SiC, even under SHI irradiation at high‐temperature (≈ 770 K).^[^ ^40^ ^]^ In other words, recovery of the amorphous layer thickness does not occur in a‐SiC, as is observed in a‐Ge (current study). Even in a‐Si,^[^ ^28^ ^]^ which is another important member of this elemental semiconductor's family, ionization‐induced recovery seems to be restricted at the buried a‐c interface. In contrast to both a‐Si and a‐SiC, both processes are operative in a‐Ge, with recovery over the remaining damage profile becoming active only after the amorphous layer thickness has decreased to a nearly Gaussian profile (i.e., critical thickness). Under further ionizing irradiation (for *Φ* ≥ *Φ*_0_), this remaining amorphous/defective layer undergoes continued recrystallization and defect recovery processes over the entire damaged thickness. This suggests very efficient recovery processes occur during highly ionizing irradiation in a‐Ge, not previously reported in Ge or other materials. In other words, the observed ionization‐induced recrystallization of a‐Ge seems to be peculiar only for Ge.

**Figure 7:**
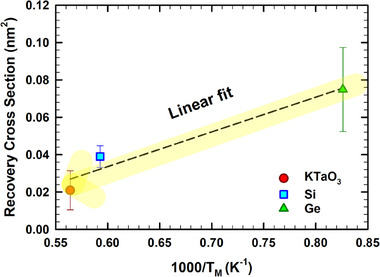
The recovery cross section (*σ*_r_) associated with ionization‐induced recovery process observed, under 12 MeV O ions irradiations, in for predamaged (not amorphized) Ge, Si,^[^ ^18^ ^]^ and KTaO_3_^[^ ^35^ ^]^ as a function of the inverse of melting temperature (*T*_M_). The dashed curve represents the best linear fit to the data. It is worth noting that the value of *σ*_r_ for all materials were the ones derived from the change of the disorder fraction at the damage peak.

**Figure 8:**
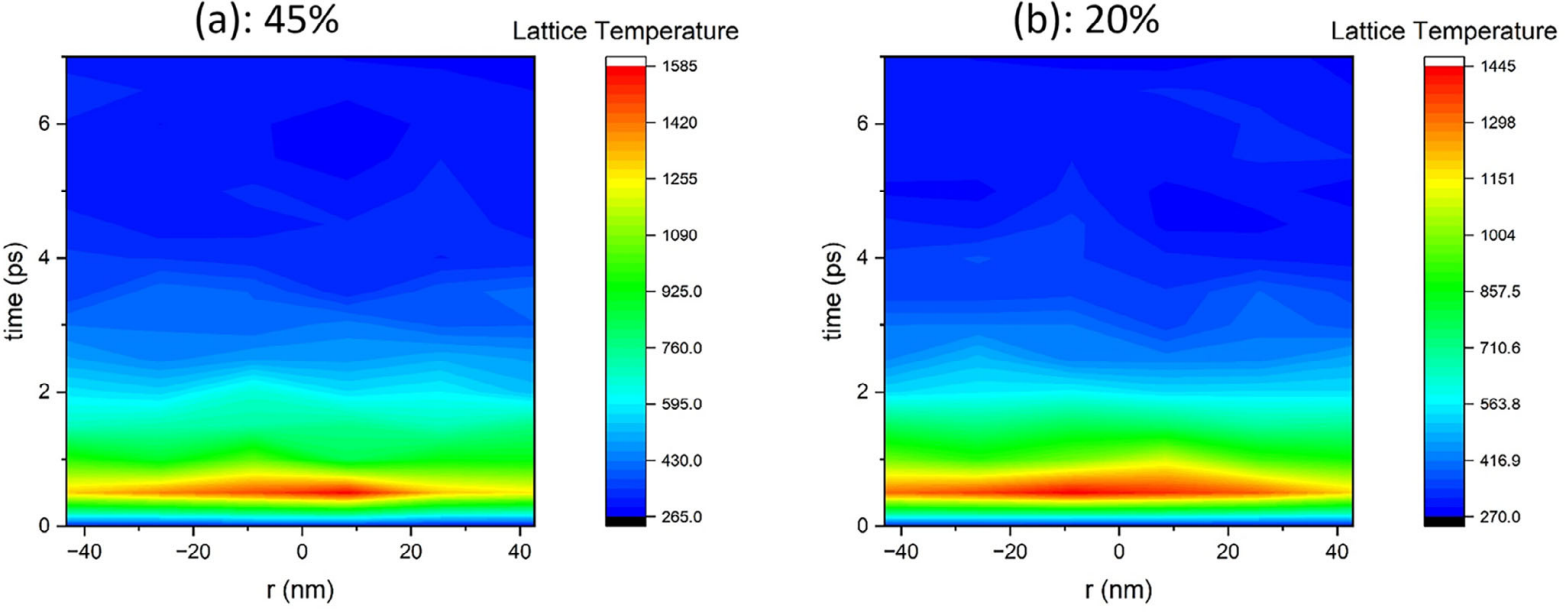
Contour maps after irradiation of the systems with a) 45% defects initial disorder and b) 20% defects initial disorder with one ion.

The derived athermal recovery cross section (*σ*_r_), from the change of the disorder fraction at the damage peak, for predamaged (not amorphized) Ge is plotted against inverse of melting temperature (*T*_M_) in **Figure** **7**, together with the *σ*_r_ associated with ionization‐induced recovery process observed in Si^[^ ^18^ ^]^ and KTaO_3_^[^ ^35^ ^]^ with *f*_0_ ≈ 0.7 and ≈0.8, respectively, and sequentially irradiated under similar conditions (i.e., 12 MeV O ions with similar ion fluence and flux). This representation reveals a monotonic increase in *σ*_r_ with decreasing *T*_M_ (and, hence, increased relative defect mobility). Here, one should note that the *T*_M_ values employed are taken from manufacturer data sheets (https://www.alineason.com/). The dashed straight line is a linear fit to the data, revealing a potential universal relationship between *σ*_r_ and *T*_M_ for some materials. This may be a further step on the way to predict *σ*_r_ in similar semiconductors susceptible to ionization‐induced athermal recovery. In summary, the average cylindrical regions centered on the O ion trajectory, where ionization‐induced annealing occurs, exhibit an inverse dependence on *T*_M_. This is consistent with the findings of Decoster et al.,^[^ ^7^ ^]^ who found a correlation between *T*_m_ and the threshold recrystallization temperature for amorphous Ge and amorphous Si. In addition to *T*_M_, the contribution of temporal thermal spike duration and the stored energy of defects and amorphous material in the systems should not be neglected because the former parameter defines the thermal spike's lifetime during which atomic vibrations/motion occur; while the latter triggers atomic motion as the stored energy is released. In **Figure** **8**, we provide the contour maps of time and temperature of the two systems, after irradiation with one ion. The plot on the left corresponds to the system with 45% predamage and the one on the right corresponds to the system with 20% predamage. The maps show the spatiotemporal evolution of ionic temperature along the *y*‐direction at the center of the *xz*‐plane, revealing rapid heating and subsequent relaxation over time following ion irradiation. Here, both the temperatures and the thermal spike durations are shown. In both cases, it is revealed that the melting temperature and sufficient annealing temperatures are reached over short timescales. Thus, the melting temperature is expected to be reached for a short time also in a‐Ge.

## 4 Summary

Understanding of athermal ionization‐induced recovery has been advanced by the discovery of substantial healing of pre‐existing collisional defects and restoration of structural order in germanium (Ge) from energy transferred to electrons via inelastic (electronic) processes. By combining experiments and modeling, this study reveals that the energy transfer of only 2.4 keV nm^−1^ from 12 MeV O ions to electrons can annihilate quite effectively the pre‐existing defects and restore the pristine Ge crystal structure at RT. These results identify a nonthermal pathway for complete structural restoration in semiconductor Ge, which could accelerate the adoption of nonequilibrium ion beam techniques in semiconductor technology for commercial devices. Spectacularly, we reveal that the irradiation‐induced c/a transformation in Ge is reversible, a phenomenon previously considered unattainable without additional thermal energy imposed during irradiation. These findings demonstrate the important effects of energy transfer to electrons in controlling c/a transformation and have broad implications across materials science, radiation damage mitigation, and fabrication of Ge‐based‐devices. Note that keeping a low flux of energetic ions, as in this study, may be a cost barrier for industrial adoption, but much higher fluxes could be utilized. Finally, we also present a comparison of *σ*_r_ associated with ionization‐induced recovery process observed in Ge, Si and KTaO_3_, which shows a monotonic increase in *σ*_r_ with decreasing melting temperature (and, hence, increased relative defect mobility).

## 5 Experimental and Modeling Sections

### Irradiation and Characterization Details

Four monocrystalline Ge samples cut from an undoped <100>‐oriented Ge wafer (dia. 2 inch × 0.42 mm thickness) with a single‐sided polish were used in this study following a two‐step experimental procedure. The samples were ≈10 × 10 mm^2^ in size. A thick clamp was used to both fix the sample and to preserve a small virgin area (1.2 × 1.2 mm^2^), as reference for later alignment (channeling coordinates). For all XRD experiments, the 0.2 mm×10 mm X‐ray beam was positioned in the center of the samples; in such a way to exclude the virgin area. First, three distinct predamaged states with a shallow range distribution (< 800 nm) were initially introduced in two undamaged Ge single crystals via irradiation at RT with 2.0 MeV Au ions to ion fluences of 0.03, 0.05 and 0.1 ions nm^−2^, respectively. At these indicated ion fluences, two different morphologies of damage structure are expected: amorphous pockets surrounded by distorted crystalline regions and a thick amorphous layer in the samples irradiated to 0.03 and 0.1 ions nm^−2^, respectively. Second, the separate response of the predamage states to *S*_e_ was evaluated by consecutive irradiation with intermediate‐energy incident ions (12 MeV O) at RT. It should be noted, that for 12 MeV O ions, *S*_e_‐associated processes, primarily ionization, are dominant within the predamaged surface layers of Ge, while the contribution of the *S*_n_‐induced damage production increases near the end of range (see Figure 1a). For reference, a second virgin (undamaged) Ge sample was also irradiated with only 12 MeV O ions at the same time. All ion irradiations were carried out at RT and off the main channeling direction (7° off <100> direction) to hinder ion channeling effects. A low average particle flux was used during the Au (≈1.6 × 10^10^ cm^−2^ s^−1^) and O (≈5 × 10^10^ cm^−2^ s^−1^) ion irradiations to avoid beam heating. The ionization‐induced evolution of pre‐existing defects in Ge was evaluated by ex‐situ RBS/C using 2 MeV He ions and a Si detector positioned at a backscattering angle (155° with respect to the incoming beam direction) for detecting the backscattered 𝛼 particles. The ion irradiations and RBS/C measurements were performed using the 3 MV Tandetron Cockcroft–Walton accelerator located at “Horia Hulubei” National Institute for Physics and Nuclear Engineering (IFIN–HH), Magurele, Romania.^[^ ^41^, ^42^ ^]^ The *S*_n_ and *S*_e_ values were calculated using the stopping and range of ions in matter (SRIM‐2003) code^[^ ^43^ ^]^ in full cascade mode with a density of 5.323 g cm^−3^ that is reported by the manufacturer (https://www.alineason.com/). The corresponding local damage dose in displacements per atom (dpa) was also calculated, for the Au ion fluences used in this study, via SRIM using as input the threshold displacement energy reported in literature, 15 eV.^[^ ^7^, ^44^, ^45^ ^]^ The SRIM predicted *S*_e_ and *S*_n_ energy loss for 12 MeV O ions in Ge along with predicted dpa profile for a fluence of 0.03 Au^−^ nm^−2^ are illustrated in Figure 1a. In this figure the shaded region (i.e., 0 *< z <* 1000 nm) represents the RBS/C characterization region. Quantitative evaluation of relative Ge disorder for the predamaged Ge prior to and after subsequent 12 MeV O irradiation necessitates extracting the disorder profiles (i.e., relative disorder vs depth) from the RBS/C spectra. The relative disorder profile corresponding to each ion fluence is derived by normalizing the corresponding RBS/C spectrum relative to the amorphous (random) and pristine (undamaged) spectra and subtracting the fraction of analyzing He ions that are dechanneled. Since ion channeling measurements conducted for the O irradiated alone samples confirm that a non‐negligible damage buildup within the first micrometer is formed for O fluence above 355 ions nm^−2^ (see Figure S1b, Supporting Information), the corresponding ion channeling spectra for O irradiated alone samples, only for fluence ≥ 355 ions nm^−2^, are used for normalization instead of using the undamaged spectra. More specifically, this iterative procedure determines the relative probability of scattering between the analyzing He ions and the displaced atoms and lattice distortion (e.g., caused by extended defects) that are directly proportional to the lattice “‘imperfections.”’ In this analysis, Ge is amorphized if the magnitude of the relative disorder is equal to 1.0; whereas for the pristine crystal (undamaged), it is presumed to be 0. The depth scale (nm) of the profile is depicted from the energy corresponding to each channel and the SRIM‐predicted stopping power for He ions in Ge. The curve fits to the data (solid lines) are used to extract the peak relative disorder following each incremental O ion irradiation fluence.

A Bruker D8 “Discover” X‐ray diffractometer was used to perform XRD measurements. The X‐ray beam from a copper target was collimated using a parabolic multilayer mirror and a four‐reflection Ge (220) monochromator tuned to select the Kα_1_ radiation of the target (*ʎ* = 1.5406 Å). The diffracted *x*‐rays were collected using a 1D position sensitive detector (“Lynx Eye”) with a resolution of 0.01° 2*θ*. *θ*‐2*θ* scans across the 400 Bragg reflection of Ge have been carried out with an angular range wide enough to include all the signal diffracted from the irradiated region. When possible, the data have been fitted with RaDMaX‐online^[^ ^30^ ^]^ in order to extract the strain and disorder depth profiles (DW) in the irradiated region.

### Modeling Methods

The DL_POLY MD code^[^ ^46^ ^]^ has been used to perform the MD simulations, using the Tersoff potential for Ge.^[^ ^47^ ^]^ The size of each Ge simulation cell was chosen to be sufficiently large to contain the equivalent increment of electronic energy deposited by 12 MeV O ions, and this was 10 nm × 10 nm × 5 nm. The pre‐existing damage in the systems was introduced by creating different concentrations of Frenkel pairs (FPs) and then performing equilibration of the systems under the NPT ensemble (constant number of atoms (N), pressure (P) and temperature (T)) at 300 K with 1 fs timestep for 60 ps. The Frenkel pairs were introduced in the system randomly using the Atomsk code.^[^ ^48^ ^]^ Different levels of defects were generated starting from 5% to 60% defects with steps of 5% or 10%. In each case the system was allowed to relax for 50–100 ps before adding more defects. During the relaxation dynamics the initially introduced level of defects somewhat changes, hence resulting with structures with 20% and 45% defects predamage. The defects are identified using the sphere criterion, with a cut‐off radius of 0.75 Å.^[^ ^18^, ^47^, ^49^, ^50^ ^]^ The two‐temperature model (2T‐MD) was employed to perform the irradiation simulations, which is suitable to simulate irradiation simulations in metals,^[^ ^49^, ^51^, ^52^, ^53^ ^]^ semiconductors, or insulators,^[^ ^18^, ^54^, ^55^ ^]^ where the energy from the fast‐moving projectile is transferred to the atomic subsystem via electron–phonon coupling. 1200 ions were used in each system, with the ion path along the *z* direction of the MD box. The interval between irradiation events was 7 ps, which was long enough for the system to cool down after each event. The electronic diffusivity temperature dependance is described by $D_{e}\;\left(T\right)=D_{0}\;\frac{T_{0}}{\min(T,T_{f})}$ as described in,^[^ ^46^ ^]^ where *D*_0_ is the value at room temperature (65 cm^2^ s^−1[^ ^56^ ^]^), and *T_f_* is the Fermi Temperature. The electron–phonon relaxation time was taken to be 0.54 ps.^[^ ^57^ ^]^ It was previously shown that the e‐ph coupling parameter (and hence, the e‐ph coupling time) increases as defects, which act as scattering centers, are introduced in the system and that this is a significant parameter in irradiation, affecting the energy dissipation in the system.^[^ ^58^ ^]^ Here it was chosen to use the pristine e‐ph coupling parameter to capture the minimum effect of annealing and account for the fact that as the damage increases the relaxation time increases (weakening e‐ph coupling). Using a shorter relaxation time (stronger e‐ph coupling) would likely enhance the annealing at the beginning of the simulation when the damage level is higher, but it will not account for the decreasing damage (increasing relaxation time) as the annealing is taking place.

## Conflict of Interest

The authors declare no conflict of interest.

## Author Contributions

G.V. conceived the project, G.V. and W.J.W. supervised the project. D.I. performed ion irradiations and ion channeling measurements. E.Z. performed theory and simulation work. A.B. performed XRD measurements and analyzed them. Y.T. and D.C. performed HAADF analysis. M.D.M. performed the partial analysis of ion channeling data. G.V., W.J.W., and E.Z. prepared the original paper. Y.Z., Y.T., and A.B. reviewed and edited the original paper. All authors discussed the results.

## Supporting information



Supporting Information

Supplemental Movie 1

Supplemental Movie 2

## Data Availability

The data that support the findings of this study are available from the corresponding author upon reasonable request.
