# Supplementary material for: Revealing a Pathway for Low‐Temperature Recrystallization in Germanium
Source: Adv Sci (Weinh). 2025 Aug 14;12(41):e07630. doi: 10.1002/advs.202507630 (PMC12591139; doi:10.1002/advs.202507630)
Supplement: Supplementary file 1 — Supporting Information [file ADVS-12-e07630-s003.docx]

Supplementary Material

**Revealing a Pathway for Low-Temperature Recrystallization in Germanium**

Gihan Velişa^a*^, Eva Zarkadoula^b^, Decebal Iancu^a^, Maria Diana Mihai^a^, Alexandre Boulle^c^, Yang Tong^d^, Da Chen^e^, Yanwen Zhang^f^, William John Weber^g**^

^a^Horia Hulubei National Institute for Physics and Nuclear Engineering, Măgurele, IF 077125, Romania

^b^Center for Nanophase Materials Sciences, Oak Ridge National Laboratory, Oak Ridge, TN 37831, USA

^c^IRCER, CNRS UMR 7315, Centre Européen de la Céramique, 12 rue Atlantis, 87068 Limoges Cedex, France

^d^Institute for Advanced Studies in Precision Materials, Yantai University, Yantai, Shandong264005, China

^e^School of Energy and Environment, Southeast University, Nanjing, China

^f^Department of Mechanical and Materials Engineering, Queen’s University, Kingston, Ontario, K7L2N8, Canada

^g^Department of Materials Science & Engineering, University of Tennessee, Knoxville, TN 37996, USA

**RBS/C results**

Fig. S1 (a) shows that following irradiation with 0.1 Au nm^-2^, the highest fluence employed in the present study, produces a sharp increase in damage up to the random level as measured by RBS/C, consistent with the generation of a continuous amorphous layer in Ge ^[1]^. It is evident that sequential irradiation with 12 MeV O ions at fluences of < 130 ions nm^-2^ leads to a continuous reduction in the amorphous layer thickness with O fluence. However, from Fig. S1 (a), a fluence of 130 O^+^ nm^-2^ also reveals an initial decrease in the peak level of disorder as measured by ion channeling. The yield of backscattered ions at the damage peak exhibits a systematic decrease with continued irradiation to ion fluences from 130 to 750 ion nm^-2^ (the highest O fluence employed in the present experiments), which is consistent with the defect annealing generated by the electronic energy loss (S_e_) component of the irradiating 12 MeV O. The RBS/C spectra plotted in Fig. S1 (b) reveal that irradiation of pristine Ge with 12 MeV O ions at fluences of < 500 ions nm^-2^ generates small, but measurable, amount of disorder. At a fluence of 750 nm^-2^, there is a clear increase in disorder.

**High-angle annular dark field (HAADF) results**

To validate that 12 MeV O ions irradiation can be used as a room-temperature approach to anneal pre-existing defects and repair the structural order, high-angle annular dark field (HAADF) analysis was carried out. Fig. S2 (a) and (b) show HAADF images of a pre-damaged Ge sample with *f_0_* ~ 0.59, before and after 12 MeV O ions irradiation to an ion fluence of 40.0 O^-^ nm^-2^, respectively. Both images were recorded at the same depth (∼250 nm below surface, Au-induced damage peak).  Here it is important to note that the contrast in Fig. 2(a) is higher compared with Fig. 2 (b); however, the contrast in Fig. 2 (b) is rather uniform. Note that the brightness of each atomic column is roughly proportional to the square of the average atomic number (Z^2^) of the column. The high contrast in Fig. 2 (a) indicates a high variation of atomic density in each column. This finding indicates that Ge samples are very sensitive to the the focused ion beam (FIB) technique used to prepare TEM specimens. TEM sample preparation using a tripod polisher technique should be taken into consideration for future samples.

We also provide the following files as Supplementary Information:

1. Movie of 12 MeV O irradiation in Ge with 20 % FPs pre-damage level.
2. Movie of 12 MeV O irradiation in Ge with 40 % FPs pre-damage level.


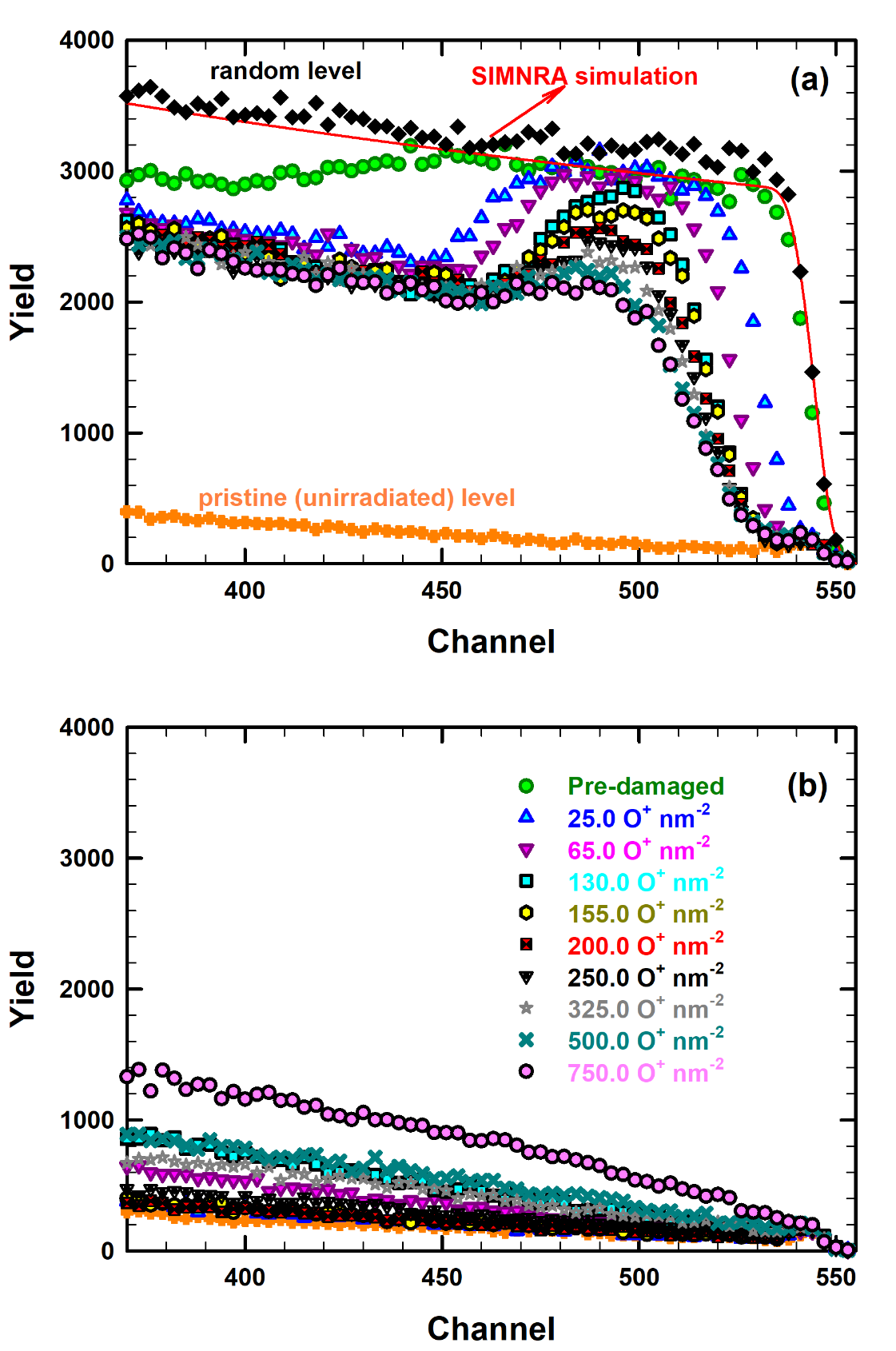


**Fig. S1**. Several RBS/C spectra, after irradiation with 12 MeV O for: (a) pre-amorphized Ge using 2 MeV Au ions to a fluence of 0.1. ions nm^-2^, and (b) pristine Ge without pre-damage. Random and channeling spectra from a pristine Ge sample are also included in (a), and a best fit obtained with SIMNRA software ^[3]^ is superimposed on the random spectra (see the red solid line).


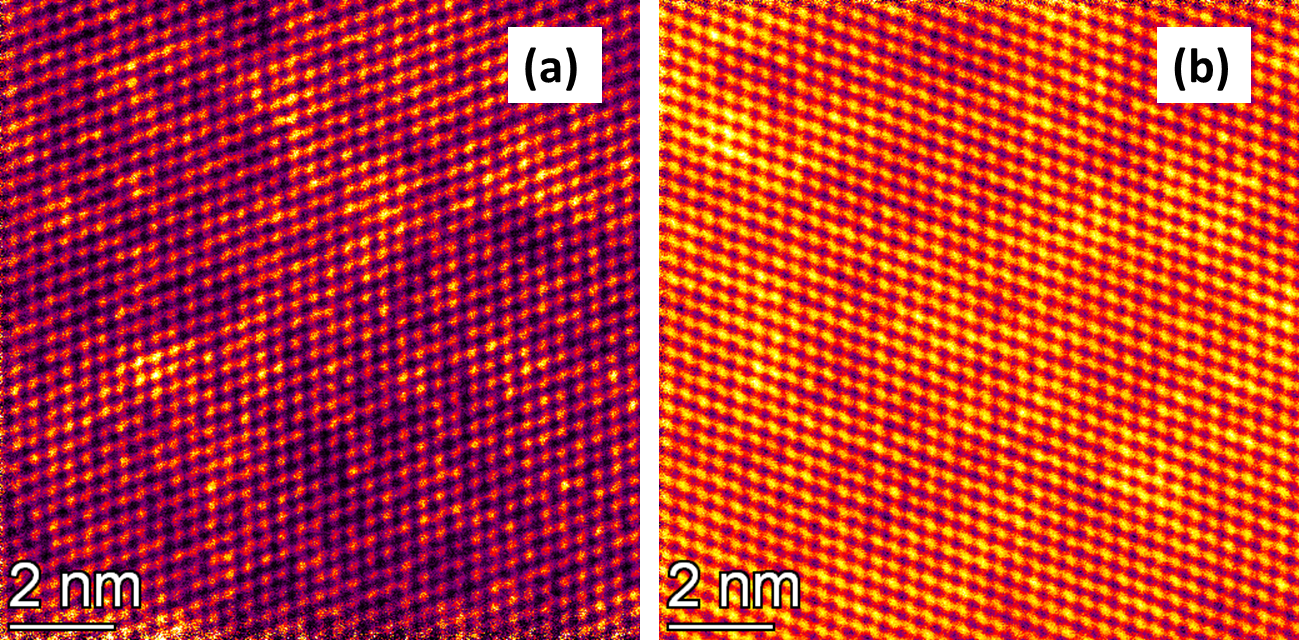


**Fig. S2**. HAADF micrographs illustrating the atomic-level microstructure changes of the Ge single crystals: (a) pre-damaged with 2.0 MeV Au ions to ion fluence of 0.03 Au^-^ nm^-2^ at 300 K and (b) subsequently irradiated with 12 MeV O ions to ion fluence of 40.0 ions nm^-2^ at 300 K. Note that the HAADF micrographs were recorded at the Au-induced damage peak (~250 nm).

References

[1] S. Decoster, A. Vantomme, *Implantation-induced damage in Ge: strain and disorder profiles during defect accumulation and recovery*, Vol. 42, IOP Publishing **2009**.

[2] D. Iancu, E. Zarkadoula, M. D. Mihai, C. Burducea, I. Burducea, M. Straticiuc, Y. Zhang, W. J. Weber, G. Velişa, *Scr Mater* **2023**, *222*, 115032.

[3] M. Mayer, *Nucl Instrum Methods Phys Res B* **2014**, *332*, 176.
